# Latest Trend of Milk Derived Exosomes: Cargos, Functions, and Applications

**DOI:** 10.3389/fnut.2021.747294

**Published:** 2021-10-29

**Authors:** Xin Feng, Xiaolin Chen, Xucan Zheng, Hui Zhu, Qien Qi, Shen Liu, Huihua Zhang, Jianwei Che

**Affiliations:** ^1^School of Life Science and Engineering, Foshan University, Foshan, China; ^2^Foshan Nanhai Poultry Breeding Co., Ltd., Foshan, China; ^3^Department of Orthopaedics, Bethune International Peace Hospital, Shijiazhuang, China

**Keywords:** milk, exosome, cargo, function, application

## Abstract

Exosomes are nanosized phospholipid bilayer vesicles released to the extracellular environment. Exosomes from various tissues or cells are being studied and there has been a growing interest in milk exosomes research due to their emerging role as messengers between cells and the fact that it can be produced in large quantities with rich source of milk. Milk derived exosomes (MDEs) contain lipids, microRNAs, proteins, mRNAs as well as DNA. Studies of exosome cargo have been conducted widely in many research areas, especially exosomal miRNAs. In this paper, we reviewed the current knowledge in isolation and identification, cargos, functions mainly in intestinal tract and immunity system of MDEs. Its application as drug carriers and diseases biomarker are also discussed. Furthermore, we also consider critical challenges of MDEs application and provide possible directions for future research.

## Introduction

Extracellular vesicles (EVs) are lipid bound vesicles secreted into the extracellular space by cells. Based on the biogenesis, release pathways, size, content, and function, EVs are differentiated into three subtypes, including microvesicles, exosomes, and apoptotic bodies (1). Exosomes are nanosized (40–100 nm diameter) phospholipid bilayer vesicles released to the extracellular environment through multivesicular bodies after budding with the plasma membrane (2). Compared with other extracellular vesicles (EVs), cargos from exosomes are sorted in a regulated, non-random way and play essential roles in cell-to-cell communication (3). MDEs are regarded as one of the most important signalsomes mediating cellular communication between mother and her offspring.

Milk exosomes have been successfully separated from bovine colostrum and milk (4), porcine milk (5), rat milk (6), goat milk (7), wallaby milk (8), and human breast milk (9) (Table 1). The membrane structure of the exosome is crucial to the cargos inside. Benefiting from the phospholipid bilayer protection, separated exosomes are stable in terms of size and biological activities when stored frozen (−80°C) (38). The membrane allows miRNAs within exosomes to avoid degradation in the gastrointestinal tract and to be further absorbed in the intestine (38, 39).

**Table 1:** Summary of the isolation and validation techniques of milk derived exosomes in literature published since 2017.

**Sample Source**	**Isolation Strategy**	**Validation**	**Comments**	**Reference**
Human breast milk	Centrifugation with ExoQuick		Exosomal miR-148a was negatively associated with infant weight, fat mass, and fat free mass, while miR-30b was positively associated with infant weight, percent body fat, and fat mass at 1 month.	Shah et al. (10)
Human breast milk	Ultracentrifugation	NTA, TEM, Western blot (Hsp70, CD9)		Chen et al. (11)
Human breast milk	Centrifugation with exosome isolation kit	TEM, NTA, Western blot (CD81, CD63)	Exosomes prevent necrotizing enterocolitis by reducing inflammation and injury as well as restoring tight junction proteins.	He et al. (12)
Cow milk	Ultracentrifugation and size exclusion chromatography	TEM, Western blot (Tsg101, Flot-1, Alix, CD63)	Milk exosomes can be taken up by intestinal epithelial cells and mediated functional intracellular delivery of siRNA.	Warren et al. (13)
Cow milk	Ultracentrifugation and size exclusion chromatography	Western blot (Hsp 90, CD63, Tsg101), NTA	Milk exosomes can be sued as nanocarriers of functional miRNAs in RNA-based therapy.	Pozo-Acebo et al. (14)
Cow milk	Ultracentrifugation	DLS, SEM, Western blot (CD63, CD9)	Milk exosomes attenuated purine nucleotide catabolism and improved energy status in oxidatively stressed IEC-6 cells	Wang et al. (15)
Cow milk	Ultracentrifugation	DLS, TEM, Western blot (CD9, CD63)	Exosomes successfully delivered epicatechin gallate into SHSY5Y cells and exhibited enhanced neuroprotective effects.	Luo et al. (16)
Cow milk and colostrum	Ultracentrifugation	Western blot (CD9, CD63milk only, Hsp70)	Exosomes can be taken up by human intestinal epithelia cells, not cytotoxic	Ross et al. (17)
Cow milk and yak milk	Ultracentrifugation with rennet precipitation	TEM, DLS, Western blot (CD63, Hsp70, Tsg101)	Milk exosmes alleviated LPS induced intestinal inflammation by inhibiting PI3K/AKT/C3 pathway activation.	Gao et al. (18)
Porcine colostrum and mature milk	Ultracentrifugation and SEC	NTA, TEM, Western blot (Tsg101)	The pathways related to homeostasis are upregulated in colostrum exosomes whereas pathways related to endothelia cell development and lipid metabolism are upregulated in milk exosomes.	Ferreira et al. (19)
Caprine colostrum and mature milk	Ultracentrifugation		miR-30a-5p, miR-22-3p, and miR-26a are highly conserved in colostrum and mature milk in cows, caprines and humans.	Yun et al. (20)
Colostrum powder	Ultracentrifugation	DLS, NTA, AFM	Exosomes can reduce or completely mitigate the immunotoxicity effects caused by solvent based paclitaxel.	Kandimalla et al. (21)
Porcine colostrum and mature milk	Ultracentrifugation and SEC	NTA, TEM, Western blot (Tsg101)	The pathways related to homeostasis are upregulated in colostrum exosomes whereas pathways related to endothelia cell development and lipid metabolism are upregulated in milk exosomes.	Ferreira et al. (19)
Cow milk	Ultracentrifugation	DLS, SEM, Western blot (CD63, CD9)	Milk exosomes attenuated purine nucleotide catabolism and improved energy status in oxidatively stressed IEC-6 cells	Wang et al. (15)
Human breast milk	Centrifugation with ExoQuick		Exosomal miR-148a was negatively associated with infant weight, fat mass, and fat free mass, while miR-30b was positively associated with infant weight, percent body fat, and fat mass at 1 month.	Shah et al. (10)
Human milk	Ultracentrifugation	NTA, TEM, Western blot (Hsp70, CD9)		Chen et al. (11)
Human breast milk	Centrifugation with exosome isolation kit	TEM, NTA, Western blot (CD81, CD63)	Exosomes prevent necrotizing enterocolitis by reducing inflammation and injury as well as restoring tight junction proteins.	He et al. (12)
Cow milk	Ultracentrifugation	DLS, TEM, Western blot (CD9, CD63)	Exosomes successfully delivered epicatechin gallate into SHSY5Y cells and exhibited enhanced neuroprotective effects.	Luo et al. (16)
Skim milk	Ultracentrifugation	DLS, TEM, Western blot (CD9, Tsg101, CD63)	Exosomes and miRNA can cross the placenta and promote embryo survival in mice	Sadri et al. (22)
Cow milk	Differential centrifugation	DLS, Western blot (Tsg101, CD81, Alix)	Milk exosomes can protect macrophages from chemotherapeutic drug-induced cytotoxicity.	Matic et al. (23)
Cow milk	Ultracentrifugation		IL-2 and IL-12 enhanced IFN-γ production with cow milx exosomes but not alone.	Komine-Aizawa et al. (24)
Cow milk	Differential centrifugation	Western blot(CD9, CD63, Tsg101)	Milk exosome based drug delivery system constructed showed controlled drug release, biocompatibility and effective in treating OSCC	Zhang et al. (25)
Cow milk	Ultracentrifugation	TEM, NTA, Western blot (Tsg101, Alix, CD9, CD81)	Exosomes can alter murine gut microbiota and SCFA in feces, as well regulate local intestinal immunity.	Tong et al. (26)
Canine colostrum	Ultracentrifugation	Western blot (Alix, Tsg101, Hsp70)	Exosomes modified the proliferation and secretory profiles in canine mesenchymal stem cells.	Villatoro et al. (27)
Human Breast milk	Centrifugation and filtration with ExoQuick reagent	Electron microscopy, DLS	Human milk-derived exosomes induced proliferation- and epithelial mesenchymal transformation-related changes. MDEs inhibited proliferation and DNMT1 expression in cells with knockdown of miRNA-148a	Reif et al. (28)
Cow milk	Ultracentrifugation	DLS, TEM, Western blot (CD63, CD81, ANXA5, FLOT1, ICAM, Tsg101)	Exosomes can be absorbed as intact perticles from the gastrointestinal tract via the Fc receptor. It can be modified with ligands to promote retention in target tissues.	Betker et al. (29)
Cow milk	Ultracentrifugation	TEM, DLS, Western blot (CD9, CD63)	Milk exosomes contain lncRNA which participate in immunity, development, and reproduction. LncRNA had different expression patterns during different stages of lactation.	Zeng et al. (30)
Cow milk	Ultracentrifugation	NTA, TEM, Flow cytometry (CD63, Hsp70, CD9, CD81)	Milk exosomes prevented experimental NEC-induced intestinal injury by increasing goblet cell production and ER function.	Li et al. (31)
Yak milk and cow milk	Ultracentrifugation/ ultracentrifugation with rennet precipitation	TEM, DLS, Western blot (CD63, Hsp70, Tsg101)	Yak milk exosomes has a more efficient effect on IEC-6 cell growth under hypoxic conditions than cow milk exosomes.	Gao et al. (32)
Breast milk	Ultracentrifugation	NTA, Western blot (CD81, clathrin)	Human breast milk-derived exosomes allow IECs to be protected from oxidative stress.	Martin et al. (33)
Skim milk	Ultracentrifugation	NTA, TEM, Western blot (CD9, Tsg101, CD63)	Milk exosomes are bioavailable and miRNAs have unique tissue distribution patterns	Manca et al. (34)
Cow milk	Ultracentrifugation	DLS, NTA, SEM, AFM, Western blot (CD63, CD81, Tsg101, Alix)	Milk exosomes can deliver chemotherapeutic drug paclitaxel and effectively inhibit tumor growth.	Agrawal et al. (35)
Cow colostrum	Density gradient centrifugation	Western blot (Alix, Tsg101)	Colostrum derived exosomes are enriched with proteins regulating the immune response and growth.	Samuel et al. (36)
Buffalo milk	Centrifugation Exiqon isolation kit	SEM, DLS	As delivery vehicle, exosome encapsulation enhances the stability, solubility and bioavailability of curcumin.	Vashisht et al. (37)

*OSCC, oral squamous cell carcinoma; TEM, transmission electron microscope; NTA, nanoparticle tracking analysis; NEC, necrotizing enterocolitis; ER, endoplasmic reticulum; DLS, dynamic light scattering; SEM, sanning electron microscopy; AFM, atomic force microscopy; AFM, atomic force microscopy*.

**Figure 1:**
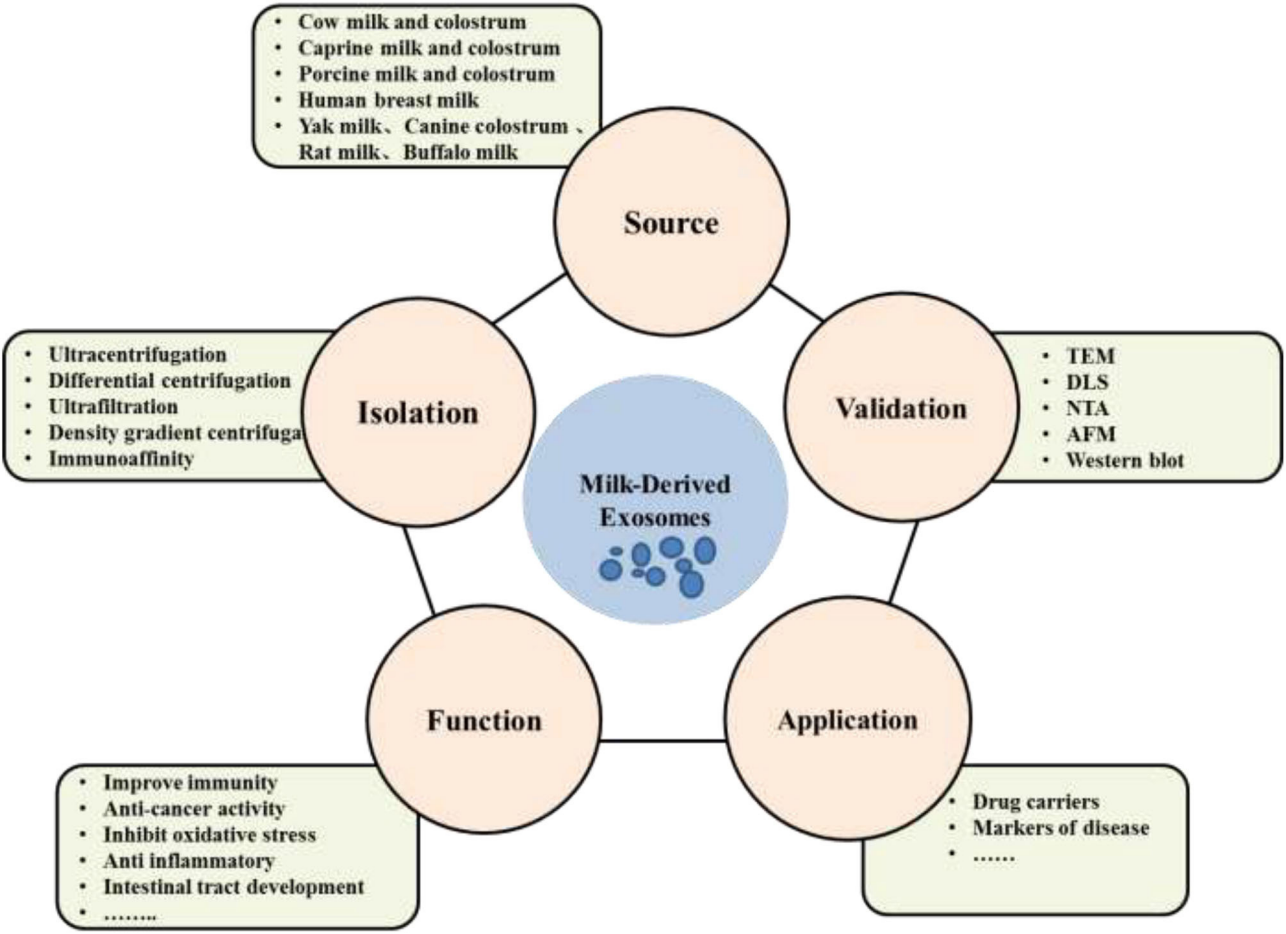
Sources of milk derived exosomes, isolation and validation methods, functions as well as applications.

The aim of this review is to comprehensively summarize and discuss the current research status of milk exosomes including isolation and identification, cargos as well as application in the scientific area. Future research and further application of MDEs is also discussed (Figure 1).

## Isolation and Identification

Ultracentrifugation is the most popular method to separate exosomes from various fluids (40). However, it requires great amount of time to complete. For example, the centrifugation with subsequent sucrose density gradient ultracentrifugation required more than 24 h (4). Ultrafiltration procedures were also used to successfully purifying exosomes and do not require special equipment (41). Combined use of membrane filters (0.1–0.2 s) with differential centrifugation could better eliminate the large vesicles and obtain relatively pure exosomes. In addition, HPLC-based method could obtain highly pure exosomes but these processes need dedicated equipment and are not easy to scale up (42). Exosomes can also be obtained by using precipitation methods, such as using polyethylene glycol, then the precipitate can be isolated using low-speed centrifugation or filtration. Commercially precipitation solutions such as ExoQuick has been used by many researchers as it increases exosome recovery and is relatively rapid (40). To obtain more specific isolation of exosomes, immunoaffinity capture methods based on affinity with antibodies to exosomal proteins or specific saccharide residues on the exosome surface have also been used by many researchers.

Exosomes are identified by size, morphology, and membrane proteins. Physical analysis are done using nanoparticle tracking analysis (NTA), dynamic light scattering (DLS), electron microscopy, and tunable resistive pulse sensing (tRPS) in order to determine particle size or concentration. Chemical or biochemical analysis are done via staining, immunoblotting, or proteomic analysis to give information on the exosomal contents (43). Regardless of source, exosomes have a buoyant density range of 1.13–1.21 g/ml. Using Transmission electron microscopes (TEM), Chen et al. (5) observed a greater density at the center of the milk exosomes by ultracentrifugation. Under TEM, MDEs normally exhibit a round morphology and uniform, unimodal distribution in size.

The exosomal proteins are heavily dependent on the tissue or cell type from which is it derived. Due to their endosomal origin, exosomes normally contain membrane transport and fusion proteins, tetraspanins, heat shock proteins, proteins involved in multivesicular body biogenesis, as well as lipid-related proteins and phospholipases (44). Due to different sources, wide variations regarding proteins exist across exosomes. Both colostrum and mature MDEs contain surface marker proteins including tetraspanins such as CD9, CD63 and CD81, milk fat globule-epidermal growth factor-factor 8 (MFG-E8), raft-associated proteins including flotillin-1 as well we internal markers such as tumor susceptibility gene 101 (Tsg101), ALG-2-interacting protein X (Alix), and heat shock protein 70 [HSP70; (45, 46). The endosomal sorting complex and other associated proteins such as Alix and Tsg101 are involved in exosome biogenesis thus are observed (47). Exosomes do not have surface markers such as integrin-β1, p-selectin, CD40, and endoplasmic reticulum (ER) marker calnexin which are considered as markers for other multivescular bodies (48). The antibody-based techniques including western blot or ELISA are popular for rapid confirmation of exosomal proteins.

## Cargos of Milk Derived Exosomes

**Figure 2:**
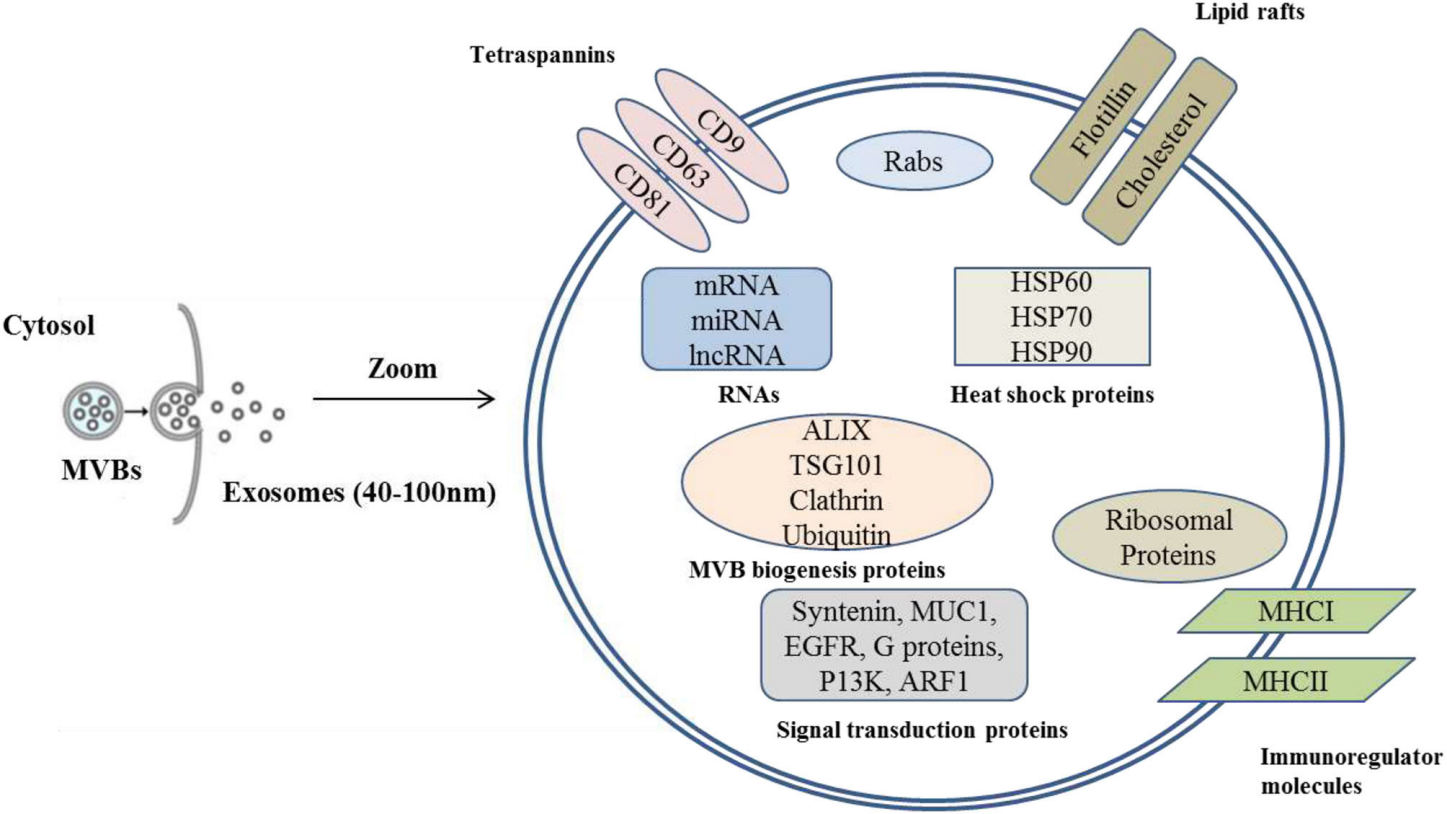
Graphical representation of milk derived exosomes showing general exosomal cargos.

Similar to exosomes isolated from other fluids or tissues, cargos of MDEs include lipids, microRNAs, proteins, mRNAs, and DNA (3, 49) (Figure 2). The exosomal cargos are sorted in a regulated, non-random process and they play essential roles in cell-to-cell communication (3).

### MicroRNAs

MicroRNAs (miRNAs) are small regulatory RNA molecules consisting of 19–24 nucleotides in length and play important roles in a wide range of physiologic and pathologic processes (50, 51). MDEs were found to contain a considerable amount of mammary gland and immune-related microRNAs. Hata et al. (4) found that RNA below 200 nt was more concentrated in the MDEs than the supernatant. Among the 24 human breast milk exosome samples, miRNAs had a range of 9.7–228.2 ng/ml (52). Porcine MDEs were reported to contain 176 known and 315 novel mature miRNAs (5). Similarly, this was also reported in bovine colostrum and milk by Hata et al. (4). Lin et al. (53) reported increased miRNAs in piglet serum by orally ingestion of bovine and porcine milk exosomes. Colostrum had higher expression of immune-related miRNAs than mature milk. Compared to piglets given in mature milk, 9 of 13 immune-related miRNAs had higher expression in the serum of piglets given colostrum (54). Izumi et al. (39) reported that immune (miR-15b, miR-27b, miR-106b, miR-155, and miR-223) and development related miRNAs (miR-27b, miR-34a, and miR-130a) are significantly higher in colostrum than in mature milk.

Several abundant exosomal miRNAs from human and porcine milk were shared between species. The conservation of the miRNAs shared among species is both in sequence homology and their incorporation in milk extracellular vesicles which indicate that they are evolutionarily selected to benefit the newborn (55). MiRNAs profiles are reported to differ among different species (39, 51). Expression of miRNAs in milk exosomes is affected by many other factors, such as host health condition and lactation stage. Up-regulation of miR-142-5p, miR-223, miR-183, and miR-99a-5p and down-regulation of miR-2285-3p and miR-101 were detected when the bovine mammary gland was challenged with *Staphylococcus aureus* infection (56). Chen et al. (51) identified 245 miRNAs in raw milk and individual miRNA can be significantly altered at different periods of lactation. Interestingly, the expression of seven miRNAs are relatively constant throughout the lactation process. Izumi et al. (39) reported that mothers' background (race and lifestyles) can also influence milk miRNAs profile.

### Proteins

Various researches have been focusing on the exosomal miRNAs. Proteins cargos also play important roles in physiology and pathology. Wang et al. (57) reported that exosomal proteins promote pre-metastatic niche formation and modulate the site-specific metastasis of tumor cells by inducing lymphangiogenesis, angiogenesis, and permeability. Exosome also delivers cargos including proteins and RNAs to the brain and it was shown that feeding an exosome- and RNA-depleted diet impaired spatial learning and memory in mice (58). The β-amyloid and α-synuclein are related to the propagation and diagnosis of Alzheimer's Disease and Parkinson's Disease and exosomes appear to participate in the spread of these two proteins (59). Rahman et al. (60) reported that proteins within milk extracellular vesicles provide information of host physiology and immunology. They observed 118 differentially expressed proteins between uninfected cattle and bovine leukemia virus-infected cattle. These proteins are involved in diverse biological activities such as metabolic processes, cellular processes, catalytic activities, and so on. Samuel et al. (36) using quantitative proteomics analysis showed that the proteomic cargo of exosomes change based on the lactation state of cows. Compared to that mature MDEs which were enriched with proteins related to transport and apoptosis, colostrum derived exosomes are more enriched with proteins implicated in the immune response, inflammatory response, antimicrobial peptides, cell growth and complement activation.

### mRNA, rRNA, and Long Non-coding RNAs (lncRNA)

Microarray analysis showed that most of the milk mRNAs were present in exosomes. Hata et al. (4) observed a considerable amount of mRNAs in milk exosomes. Admyre et al. (52) reported that ribosomal RNA (18S and 28S) is very low in human breast MDEs. Presence of exosomal mRNAs such as CD63, CD36, Eα1, FAS, MFG-E8, MHC-II were confirmed in both milk and colostrum by RT-PCR (45). Zeng et al. (30) identified 3,475 novel lncRNAs and 6 annotated lncRNAs in bovine milk exosomes. They also observed that expression of lncRNAs vary across the stages of lactation. The exosomal RNAs can exert functional effects because of their stability as they are packaged in membranes (61).

## Stability

Cargos encapsulated in exosomes membranes are protected against enzymatic and non-enzymatic degradation. MiRNAs and mRNAs were detected with a considerable amount in the milk although RNase concentration was extremely high (4). The miRNAs and mRNAs have been found in commercial dairy products, such as infant formula, which have undergone stringent industrial processes (51). These RNAs are also stable under harsh treatments including low pH, RNase, and freezing condition (4, 39). The miRNAs in breast MDEs are still high after RNase, freeze-thawing and acidic (pH = 1) treatments (62), thus allowing dietary intake of miRNAs by infants. However, synthetic miRNAs were rapidly degraded under the degradative conditions whereas the endogenous milk miRNAs were resistant to treatment (39). Commercial dairy milk is highly enriched with bta-miR-223 and bta-miR-125b. Pieters et al. (63) observed relatively small differences in expression levels of immune-related miRNAs between raw milk and commercial milk suggesting that processing did not affect most of the miRNAs in the milk. The stability of MDEs makes it highly resistant against degradative conditions in the intestinal tract, thus enabling its uptake by epithelial cells. Bovine milk exosomal lncRNAs, similar to miRNAs, were stable during *in vitro* digestion, no matter with what kind of digestive juices, such as saliva, gastric juice, pancreatic juice, and bile juice (30). Benmoussa et al. (64) simulated gastrointestinal tract conditions and tested how *Bos Taurus* bta-miR-223 and bta-miR-125b withstood digestion. The authors found out that although the miRNAs were decreased most in the stomach, a large number of miRNAs in the upper small intestine compartments suggested their bioaccessibility.

Although it was experimentally proved that the exosomal cargos were effectively protected by the exosomal membrane, the exosomal miRNAs in artificial formula are deficit compared to the raw milk (65). The pasteurization process can decrease exosomes concentrations by ~50%, due to membranes disruption and cargos degradation (66) by disrupting. The levels of miRNAs are significantly lower in the infant formula compared to raw milk, especially the extensively hydrolyzed formula (39). A loss of milk miRNAs and exosomes caused by ultra-heat treatment was observed previously (67). Ultrasonication can also affect exosome morphology and cause substantial loss in RNA cargos. In milk, miR-29b and miR-200c are among the most abundant miRNAs. Howard et al. (66) reported that pasteurization and homogenization can cause miR-200c and miR-29b loss whereas heating in the microwave caused loss of miR-29b but not miR-200c. Furthermore, they can be degraded by adding detergent or bacterial fermentation (66). Surface protein removal from exosomes can decrease the exosome uptake by intestinal and vascular endothelial cells (68). Bacterial fermentation can degrade exosome membrane by attacking on exosome proteins, resulting in exosomal miRNA degradation by RNases in the environment (69).

Other than maintaining the stability of the inside components, the lipid membrane of the exosomes also has bioactivity similar the lipids found in other cellular membranes including cholesterol, phospholipids, and sphingolipids (70). To data, limited information on bovine milk exosomal lipids has been reported.

## Functions

### Intestinal Tract

Milk exosomes play an important role in the development of the digestive tract. Many studies including in mouse models or *in vitro* cell cultures showed that bovine MDEs can enter the cytoplasm by endocytosis and then release their miRNA cargos across the basolateral membrane (59). MDEs promoted goblet cell expression indicated by increasing mucin production and trefoil factor 3 (TFF3) and mucin 2 (MUC2) (31). Chen et al. (71) observed that villus height and crypt depth of the duodenum and jejunum of mice were increased with daily administration of porcine MDEs. The possible reasons were attributed to increased expression of several transcription and proliferation factors (CDX2, IGF-1R) and reduced expression of protein p53 which regulate cell death (71). Similarly, rat milk exosomes were reported to stimulate intestinal epithelial cells' (IEC-18) viability, enhance proliferation by increasing expression of proliferating cell nuclear antigen (PCNA), and stimulate stem cell activity by increasing leucine-rich repeat-containing G-protein coupled receptor 5 (Lgr5) gene expression (6). The authors explained that MDEs travel within the intestinal epithelial cells and then can be taken up by stem cells thus enhance cell proliferation and stem cell activity. Milk exosomes can be taken up by human macrophages and vascular endothelial cells as well and it may cross the intestinal mucosa and biologically activate the response signal (32, 61). It was observed that IEC-6 cells uptake more yak MDEs than cow MDEs which may be due to the fact that the yak milk exosomes had significantly higher expression of membrane proteins than cow milk exosomes. Yak MDEs increased the survival rate of IEC-6 cells in hypoxic conditions by up to 29% whereas cow milk derived milk only had 22% (32). The possible reasons were attributed to increased expression of oxygen-sensitive propyl hydroxylase- 1 (PHD-1) and decreased expression of hypoxia-inducible factor-α (HIF- α), its downstream target vascular endothelial growth factor (VEGF), and p53 (32).

Milk exosomes and their cargo miRNAs are absorbed in the upper intestine and accumulate mainly in the liver in mice (68). Many miRNAs are involved in gut health by exerting certain physiological functions (53). Inflammatory intestinal tissues had lower expression of miRNAs which are related to intestinal goblet cell differentiation and intestinal epithelium intact. Interestingly these miRNAs are highly present in MDEs (72). As components of milk exosomes, miR-148 and miR-155 can suppress intestinal T cells and may have a preventive effect on colorectal cancer carcinogenesis (73). MiR-200b can promote intestinal epithelial cell proliferation by inhibiting the epithelial-mesenchymal transition via TGF-β (74). MiR-146b can alleviate intestinal inflammation through the NF-kB pathway in a mouse colitis model and improve epithelia barrier function (75). Infant formula is miRNA-deficient and this may have negative effects on long-term immunological and metabolic programming of the infants (69). Short chain fatty acids (SCFA) in the gut are the major energy sources of intestinal epithelial cells and promote intestinal health by increasing epithelial absorption cells. Oral administration of MDEs increased SCFA production and number of epithelial absorption cells (26). The abundance of *Lachnospiraceae* and *Ruminococcaceare* were also increased as they are associated with the maintenance of gut health. Ross et al. (17) evaluated the effects of bovine colostrum and milk derived exosomes from high, average and low responder cows on human colorectal adenocarcinoma epithelial (Caco-2) cells. They found that co-incubation with exosomes maintain Caco-2 cells metabolic activity. Metabolic activity after incubation with exosomes from high responder cows was significantly greater than that from the low responder cows. It indicates that milk from cows with different immune response genetics might have different effects on gut health. Furthermore, the author pointed out that both colostrum and milk exosomes enhanced cell viability but did not stimulate oncogenic proliferation of Caco-2 cells *in vitro*.

Exosomes can inhibit the activation of toll-like receptor 4 (TLR4) which was involved in intestinal inflammation and progression of necrotizing enterocolitis (NEC) (76). In normal colonic epithelial cells, MDEs changed the cells from classic cuboid shape to a mesenchymal-like shape and contributed to cell proliferation but this was not observed in the tumor cells (28). The underlying mechanisms were attributed to the fact that MDEs upregulated the expression of collagen type I and downregulated *twist1* gene expression and phosphatase and tensin homolog (PTEN) protein in normal colonic epithelial cells but not in tumor cells (28). Exosomes gavage also prevented the necrotizing enterocolitis by preventing the ileal morphological injury and reduction in MUC2+ goblet cells and glucose-regulated protein 94 (GRP94+) cells per villus. MiR-200a-3p is a negative regulator of the pro-inflammatory chemokine ligand 9. It was observed that feeding diet depleted of milk exosomes in Mdr1a^−/−^ mice can elicit depletion of miR-200a-3p, elevated cecal inflammation as well as chemokine ligand 9 expression (77). Mice fed regular diet had less intestinal lesions and lower score for gland hyperplasia and stromal collapse compared with the mice on the exosome-depleted diet (77). Martin et al. (33) pointed out that human breast MDEs can attenuate epithelial cell death from oxidative stress induced by H_2_O_2_ but not cycloheximide. However, the underlying mechanism is still not clear.

### Immune Function

Widely spread among eukaryotes, miRNAs represent key components of a conserved system of RNA-based gene regulation. MiRNAs play important roles in the process of cellular proliferation and differentiation, tissue development and differentiation, and immune response (78). Chen et al. (5) identified 176 miRNAs out of 491 miRNAs target genes in transcription, immunity, and metabolism resources in porcine MDEs. The top 14 miRNAs participate in regulation of the IgA immune network and target about 20 immune-related genes such as CD40, CD80, MADCAM1, SLA, and among others. The immune-related miRNAs are similar in cow milk and breast milk (51). Compared to serum, all of the immune-related miRNAs such as miR-181a, miR-155, and miR-223 were abundantly expressed in milk, especially in colostrum, indicating that they may play a critical role in the biogenesis and development of immune system in infants (51). The higher immune-boosting effects of colostrum compared with mature milk may be correlated with higher levels of immune-related miRNAs and gene transcripts (52).

The milk derived EVs can influence the milk recipients' immune system with the immune-regulatory miRNAs present (63). Exosomes affect intercellular communication through exosomal surface antigens with target cell receptors or via transferring exosomal RNAs and proteins to target cells (79). Under inflammatory condition, TGF-beta is required for inducing the pathogenic T-helper17 cells. TGF-beta on milk derived EVs can modulate T cell differentiation and play a role in the immune system (63). MDEs were reported to effectively alleviate the inflammatory response (increase in anti-inflammatory cytokine GM-CSF) caused by lipopolysaccharide (LPS). The lung and liver NF-*k*B levels were reduced by 30–40% suggesting the cargos inside exosomes such as immune factors, miRNAs and proteins could be exerting these protective effects (45). The expression levels of immune-related miRNAs in the first 6 months of breast milk are high (62). Breast MDEs were reported to increase the number of Foxp3+, CD4+ CD25+ regulatory T cells in infants (52) and induce B-cell differentiation (62). Milk exosomes can effectively prevent allergy of infants and they are critical for the maturation of the immune system during early infancy (52, 73). Bovine milk miRNAs can affect gene expression in peripheral blood mononuclear cells in human volunteers (68). Oral gavage of MDEs increased gene expression of Muc2, GATA4, RegII-γ, and MyD88 (gene linking gut microbiota and intestinal immunity) which are all related to intestinal immunity (26). Hata et al. (4) reported that RNAs from bovine milk-derived microvesicles might involve in the development of calf's gastrointestinal and immune systems after being transferred to living cells. The authors observed that acid treatment of milk did not drain the miRNAs, so it is possible that the miRNAs can reach innate and acquired immune cells in gut-associated lymphoid tissues of suckling calves. Izumi et al. (61) observed that bovine milk exosomes were incorporated into differentiated human monocytic leukemia THP-1 cells by using flow cytometry and fluorescent microarray techniques and the results indicated that the exosomes might affect human cells through the RNA contents. In addition, cow milk exosomes modulate immunity-related disease possibly by the methylation of cells through miRNA transfer (80, 81). Naqvi et al. (82) demonstrated that higher content of miRNA-30b can inhibit phagocytosis in myeloid inflammatory cells.

### Others Functions

Other than effects on intestinal and immune functions widely studied, milk exosomes also have beneficial effects on other areas. In human cells and in mice, bacteria invasion can induce the ADAM10 bearing exosome secretion and the exosomes can serve as decoys to bind bacterially produced toxins thus to protect the host cells. Keller et al. (83) demonstrated that ATG16L1 and other ATG proteins can provide protection against α-toxin through exosomes by releasing ADAM10. Arntz et al. (84) reported that oral delivery of bovine milk derived extracellular vesicles can ameliorate experimental arthritis in IL-1 receptor antagonist^−/−^ and DBA1/J mouse models. Skeletal muscle growth and development can also be regulated by milk exosomes. Milk exosomal miRNAs such as miR21 and miR29a can enhance muscle protein synthesis by amplifying mTOR signaling pathway (85). Spatial learning and memory depends on purinergic receptor signaling (87), Manca et al. (34) observed that exosome and their cargos can also accumulate in the brain which may explain why that dietary depletion of milk exosomes can impair spatial learning and memory due to the aberrant metabolism of purines. Furthermore, in bones, bovine milk exosomes were observed to increase osteocyte number and woven bone formation, promoting osteoblast differentiation in mice (88). Reif et al. (28) reported dual effects of miR-148a on different tumor cells: inhibiting cell proliferation in hepatocellular carcinoma and esophageal cancer whereas promoting cell growths in glioblastoma. Further studies are needed to explore the mechanisms of MDEs on different cells.

## Applications of Milk Derived Exosomes

### Drug Carriers

Many factors such as high cost, difficulty in production in sufficient quantities, and toxicity/intolerance issues have prevented the clinical introduction of many natural and synthetic materials. As ideal nanoparticles, it should have attributes including long circulation time, evasion of the host immune system, precise targeting for specific cells, minimal off site toxicity, and ability to carry versatile therapeutics (89). The application of MDEs is very intriguing and they are being explored as nanodevices for the development of new chemotherapeutic/chemopreventive carriers. Compared to other synthetic carriers, nature-derived nanoparticles such as exosomes from milk have more advantages: (1) well-tolerated in the body as it exists in various biological fluids and it exhibited cross-species tolerance with no adverse immune and inflammatory response; (2) longer circulating half-life; (3) can be internalized by other cells (90). Somiya et al. (91) demonstrated that administration of MDEs did not result in systemic toxicity and serial administration did not cause any anaphylaxis effect. Curcumin encapsulated in milk exosomes was found to resist human digestion and possess enhanced intestinal permeability due to its elevated stability, solubility as well as bioavailability (37). Another important trait for MDEs is that it can be produced in bulk with the rich source of milk. In addition, long storage had no significant changes in the physical and biological properties of milk-derived exosomes. Bovine milk exosomes are bioavailable and distribute widely among murine tissues, accumulating mainly in the liver, and spleen. Betker et al. (29) suggested that milk exosomes are absorbed from the gut as intact particles via the neonatal Fc receptor and the intact particles can be modified with ligands to retain in target tissues. For those unstable or poorly bioavailable drugs, MDEs are considered scalable vehicles to deliver them effectively. To use milk exosomes as a vehicle for drug, identifying the target tissues is important. Three hours after intravenous injection, exosome concentrations in liver and spleen peaked and then slightly decreased. In contrast, by oral gavage, the exosome signal in the liver peaked 24 h but no signal after 48 h (34).

Administration of bovine MDEs as systemic drug delivery were successful on tumor bearing mice (35). The authors found that paclitaxel loaded with exosomes inhibited tumor growth by 60% whereas paclitaxel itself only had 31%. The possible reason could be attributed to the sustained release of the drugs and the remarkably lower systemic and immunologic toxicities with the exosomes. Zhang et al. (25) developed a new milk exosome based pH/light sensitive drug delivery system and the system can control the drug release and are proved to be effective in treating oral squamous cell carcinomas (OSCC). With a bi-lipid membrane and an aqueous core, milk exosomes can be potentially used as carriers for both hydrophilic and lipophilic drugs (92). Munagala et al. (45) demonstrated that milk-derived exosomes can deliver both hydrophilic and lipophilic small molecules such as chemo drugs. In cell culture studies against lung tumor and breast cancer, drug-loaded exosomes showed significantly higher efficacy than free drug. The possible reason could be that drug loaded in exosomes had increased stability and higher cellular uptake. Furthermore, milk exosomes had no adverse immune and inflammatory response and the author suggests it can used as a biocompatible and cost effective tool to enhance oral bioavailability and improve efficacy and safety of drugs (45). Folic acid (FA) and other vitamin receptors have been extensively explored to achieve tumor targeting because normally they are overexpressed in many cancers. Milk exosomes can functionalize with tumor targeting ligand to further improve specificity and eliminate off-target side effects of drugs on healthy cells (45).

### Markers of Disease

Mastitis in dairy industry, either clinical or subclinical, has caused big economic loss due to the restraint sale of the milk from mastitis cows. Mastitis is usually caused by microbial infection, such as *staphylococci, streptococci*, and *coliform* bacteria (93). Sun et al. (56) reported higher levels of bta-miR-142a and bta-miR-223 in the milk from *S. aureus* challenged cows compared to the control cows, suggesting that these two miRNAs can be used as biomarkers of bacterial infection. Cai et al. (94) analyzed milk exosomes from three healthy and three mastitis cows and identified 18 miRNAs differently expressed between the two groups. Among the 14 differentially expressed miRNAs, nine were upregulated in the cows with mastitis (miR-142-5p, miR-142-3p, miR-103, miR-147, miR-23a, miR-223, miR-146a, miR-146b, and miR-221). The authors indicated that the differently expressed miRNAs, especially miR-223 and miR-142-5p can be used as potential marker candidates for mastitis. Chen et al. (51) suggested using seven milk-associated miRNAs (miR-26a, miR-26b, miR-200c, miR-21, miR-30d, miR-99a, and miR-148a) as potential biomarkers for the quality control of raw milk and other milk-related products. A potential biomarker for quality control in bovine milk and human milk, miR-148a, was found to be highly expressed in Yorkshire sows (51) but a moderately in Landrace pigs (5). In human, milk derived miRNAs were suggested to be used as markers to identify the novel mechanisms involved in genetic variation for breast function such as SLC20A2 (95). MiR-21, known as an oncomiR, is related to many kinds of cancer such as malignant melanoma, prostate cancer, and hepatocellular carcinoma (96, 97). The miRNAs, especially the oncomiRs deserve more investigation in diseases studies.

## Future Research

Milk exosomes could also be explored to load and deliver potentially other macromolecules such as siRNA, miRNAs, plasmid DNA, cDNA, and proteins (antioxidant enzymes, etc). Additionally, the protective effects of milk exosomes *per se* are very intriguing and suggest utility of these nanovesicles against many inflammation-based diseases. For example, exosomes from bovine milk and colostrum could be exploited as additives in formula milk and thus potentially serve as immune booster in infants and could also be used for immune-compromised cancer patients undergoing chemotherapy. Further, tissue targeting or site-specific delivery of drug loaded exosomes can be explored by adding a wide variety of tumor-targeting ligands such as antibodies (e.g., VEGF, EGFR), peptides (e.g., transferrin, integrins, Her2), or receptor-targets (e.g., FA, biotin and hyaluronic acid) to the milk exosomes.

The gut microbiota can be affected by dietary interventions and composition of microbiota change as diet changes (98). By oral gavage it was observed that 75% of exosomes not absorbed in the upper intestine enter the large intestine and cecum (34). It is noteworthy that exosomes amount entering the digestive tract can largely affect absorption as well as their interaction with gut microbiota. Understanding the biological effects of milk exosomes and their cargos on gut microbes is also intriguing and studies on effects of exosome on gut microbiota and their interactions are scarce. Oral administration of MDEs could modulate the host gut microbiota (26). Exosomes and their cargos participate in the crosstalk between gut microbes and host by altering microbial communities. A growth advantage of the bacteria cultured with milk exosome was observed *in vitro* (99). The gut microbes might act as transmitters or amplifiers of dietary exosome signals because changes in the microbial communities can cause changes in the production of microbial metabolites. Exosomes supplementation also affected hepatic concentrations of purine metabolites as well as muscle grip strength and how these phenotypes depending on the gut microbial, indirectly by exosomes, is interesting.

Genetic selection of high performance and pregnancy-dependent E2 production cause enrichment of miR-148a and miR-21. Reif et al. (28) reported upregulation of miR-148a can inhibit tumor cell proliferation. To the contrary of most reported findings, Melnik and Schmitz (73) pointed out that continuous intake of milk exosomes may pose a risk for chronic diseases including obesity, type 2 diabetes mellitus, osteoporosis, Parkinson's disease, and common cancers, mainly due to the miRNAs inside the exosomes: such as miR-148a which suppress inhibitor of adipogenesis, miR-29b which belongs the diabetogenic miR family, miR-155 which can promote the initiation and progression of Parkinson's disease in humans, and miR-21which promotes tumor progression. The miRNA-21, which was observed in milk exosomes, can enhance mTOC1-driven metabolic processes by attenuating the inhibitory effects of various tumor suppressor proteins on mTORC1-signaling (85, 86). The authors mentioned that as human breast milk is the ideal food for infant, persistent high cow milk signaling during adolescence and adulthood may promote diseases of civilization. In that paper, most of the finding are based on outcomes from consuming milk not on exosomes only, thus the negative effects of MDEs need to be further investigated.

## Author Contributions

XF, XC, HZhu, and QQ: writing. XZ, SL, and HZha: reviewing and editing. JC: artwork. All authors contributed to the article and approved the submitted version.

## Funding

The financial support from Guangdong Basic and Applied Basic Research Foundation (2019A1515110780), Discipline Construction Program of Foshan University (CGZ0400162), the research start-up fund for Postdoctoral Fellows from Foshan City (BKS209059), the Scientific research start-up fund for high-level talents of Foshan University (Gg07145), the National Natural Science Foundation of China (Grant No. 31902228), the Scientific Research Foundation in the Higher Education Institutions of Educational Commission of Guangdong Province (2017GCZX006), Guangdong Province Modern Agriculture Poultry Industry technology system innovation team construction project (2020KJ128), Guangdong Science and Technology Innovation Strategy Special Fund (DZX20192520309), and Special Foundation for Key Research Area of Educational Commission of Guangdong Province (2019KZDZX2006) were acknowledged.

## Conflict of Interest

XZ was employed by company Foshan Nanhai Poultry Co., Ltd. The remaining authors declare that the research was conducted in the absence of any commercial or financial relationships that could be construed as a potential conflict of interest.

## Publisher's Note

All claims expressed in this article are solely those of the authors and do not necessarily represent those of their affiliated organizations, or those of the publisher, the editors and the reviewers. Any product that may be evaluated in this article, or claim that may be made by its manufacturer, is not guaranteed or endorsed by the publisher.

## References

[1] Zaborowski MP Balaj L Breakefield XO Lai CPK. Extracellular vesicles: composition,biological relevance, and methods of study. Bioscience. (2015) 65:783–97. 10.1093/biosci/biv084 26955082 PMC4776721

[2] van Dommelen SM Vader P Lakhal S Kooijmans SA van Solinge WW Wood MJ. Microvesicles and exosomes: opportunities for cell-derived membrane vesicles in drug delivery. J Control Release. (2012) 161:635–44. 10.1016/j.jconrel.2011.11.021 22138068

[3] Zempleni J Lozano A Sadri M Sukreet S Manca S. Biological activities of extracellular vesicles and their cargos from bovine and human milk in humans and implications for infants. J Nutr. (2017) 147:3–10. 10.3945/jn.116.238949 27852870 PMC5177735

[4] Hata T Murakami K Nakatani H Yamamoto Y Matsuda T Aoki N. Isolation of bovine milk-derived microvesicles carrying mRNAs and microRNAs. Biochem Biophys Res Commun. (2010) 396:528–33. 10.1016/j.bbrc.2010.04.135 20434431

[5] Chen T Xi QY Ye RS Cheng X Qi QE Wang SB. Exploration of microRNAs in porcine milk exosomes. BMC Genom. (2014) 15:100. 10.1186/1471-2164-15-100 24499489 PMC4008308

[6] Hock A Miyake H Li B Lee C Ermini L Koike Y. Breast milk-derived exosomes promote intestinal epithelial cell growth. J Pediatr Surg. (2017) 52:755–9. 10.1016/j.jpedsurg.2017.01.032 28188035

[7] Chen Z Luo J Sun S Cao D Shi H Loor JJ. miR-148a and miR-17-5p synergistically regulate milk TAG synthesis via PPARGC1A and PPARA in goat mammary epithelial cells. RNA Biol. (2017) 14:326–38. 10.1080/15476286.2016.1276149 28095188 PMC5367336

[8] Modepalli V Kumar A Hinds LA Sharp JA Nicholas KR Lefevre C. Differential temporal expression of milk miRNA during the lactation cycle of the marsupial tammar wallaby (*Macropus eugenii*). BMC Genomics. (2014) 15:1012. 10.1186/1471-2164-15-1012 25417092 PMC4247635

[9] Mirza AH Kaur S Nielsen LB Storling J Yarani R Roursgaard M. Breast milk-derived extracellular vesicles enriched in exosomes from mothers with type 1 diabetes contain aberrant levels of microRNAs. Front Immunol. (2019) 10:2543. 10.3389/fimmu.2019.02543 31708933 PMC6823203

[10] Shah KB Chernausek SD Garman LD Pezant NP Plows JF Kharoud HK. Human milk exosomal microRNA: associations with maternal overweight/obesity and infant body composition at 1 month of life. Nutrients. (2021) 13:1091. 10.3390/nu13041091 33801634 PMC8066780

[11] Chen W Chen X Qian Y Wang X Zhou Y Yan X. Lipidomic profiling of human milk derived exosomes and their emerging roles in the prevention of necrotizing enterocolitis. Mol Nutr Food Res. (2021) 65:e2000845. 10.1002/mnfr.202000845 33715285

[12] He S Liu G Zhu X. Human breast milk-derived exosomes may help maintain intestinal epithelial barrier integrity. Pediatr Res. (2021) 90:366–372. 10.1038/s41390-021-01449-y 33731816

[13] Warren MR Zhang C Vedadghavami A Bokvist K Dhal PK Bajpayee AG. Milk exosomes with enhanced mucus penetrability for oral delivery of siRNA. Biomater Sci. (2021) 9:4260–77. 10.1039/D0BM01497D 33367332 PMC8205963

[14] Del Pozo-Acebo L Hazas MLL Tomé-Carneiro J Gil-Cabrerizo P San-Cristobal R Busto R. Bovine milk-derived exosomes as a drug delivery vehicle for miRNA-based therapy. Int J Mol Sci. (2021) 11:1105. 10.3390/ijms22031105 33499350 PMC7865385

[15] Wang L Wang X Shi Z Shen L Zhang J Zhang J. Bovine milk exosomes attenuate the alteration of purine metabolism and energy status in IEC-6 cells induced by hydrogen peroxide. Food Chem. (2021) 350:129142. 10.1016/j.foodchem.2021.129142 33610842

[16] Luo S Sun X Huang M Ma Q Du L Cui Y. Enhanced neuroprotective effects of epicatechin gallate encapsulated by bovine milk-derived exosomes against Parkinson's disease through antiapoptosis and antimitophagy. J Agric Food Chem. (2021) 69:5134–43. 10.1021/acs.jafc.0c07658 33890462

[17] Ross M Atalla H Karrow N Mallard BA. The bioactivity of colostrum and milk exosomes of high, average, and low immune responder cows on human intestinal epithelial cells. J Dairy Sci. (2021) 104:2499–510. 10.3168/jds.2020-18405 33358817

[18] Gao HN Hu H Wen PC Lian S Xie XL Song HL. Yak milk-derived exosomes alleviate lipopolysaccharide-induced intestinal inflammation by inhibiting PI3K/AKT/C3 pathway activation. J Dairy Sci. (2021) 104:8411–24. 34001362 10.3168/jds.2021-20175

[19] Ferreira RF Blees T Shakeri F Buness A Sylvester M Savoini G. Comparative proteome profiling in exosomes derived from porcine colostrum versus mature milk reveals distinct functional proteomes. J Proteomics. (2021) 249:104338. 10.1016/j.jprot.2021.104338 34343709

[20] Yun B Kim Y Park DJ Oh S. Comparative analysis of dietary exosome-derived microRNAs from human, bovine and caprine colostrum and mature milk. J Anim Sci Technol. (2021) 63:593–602. 10.5187/jast.2021.e39 34189507 PMC8203993

[21] Kandimalla R Aqil F Alhakeem SS Jeyabalan J Tyagi N Agrawal A. Targeted oral delivery of paclitaxel using colostrum-derived exosomes. Cancers. (2021) 13:3700. 10.3390/cancers13153700 34359601 PMC8345039

[22] Sadri M Shu J Kachman SD Cui J Zempleni J. Milk exosomes and miRNA cross the placenta and promote embryo survival in mice. Reproduction. (2020) 160:501–9. 10.1530/REP-19-0521 32621589

[23] Matic S D'Souza DH Wu T Pangloli P Dia VP. Bovine milk exosomes affect proliferation and protect macrophages against cisplatin-induced cytotoxicity. Immunol Invest. (2020) 49:711–25. 10.1080/08820139.2020.1769647 32456495

[24] Komine-Aizawa S Ito S Aizawa S Namiki T Hayakawa S. Cow milk exosomes activate NK cells and gammadeltaT cells in human PBMCs *in vitro*. Immunol Med. (2020) 43:161–70. 32649844 10.1080/25785826.2020.1791400

[25] Zhang Q Xiao Q Yin H Xia C Pu Y He Z. Milk-exosome based pH/light sensitive drug system to enhance anticancer activity against oral squamous cell carcinoma. RSC Adv. (2020) 10:28314–23. 10.1039/D0RA05630H PMC9055635 35519132

[26] Tong L Hao H Zhang X Zhang Z Lv Y Zhang L. Oral administration of bovine milk-derived extracellular vesicles alters the gut microbiota and enhances intestinal immunity in mice. Mol Nutr Food Res. (2020) 64:e1901251. 10.1002/mnfr.201901251 32180343

[27] Villatoro AJ Martin-Astorga MDC Alcoholado C Becerra J. Canine colostrum exosomes: characterization and influence on the canine mesenchymal stem cell secretory profile and fibroblast anti-oxidative capacity. BMC Vet Res. (2020) 16:417. 10.1186/s12917-020-02623-w 33138803 PMC7607682

[28] Reif S Elbaum Shiff Y Golan-Gerstl R. Milk-derived exosomes (MDEs) have a different biological effect on normal fetal colon epithelial cells compared to colon tumor cells in a miRNA-dependent manner. J Transl Med. (2019) 17:325. 10.1186/s12967-019-2072-3 31564251 PMC6767636

[29] Betker JL Angle BM Graner MW Anchordoquy TJ. The potential of exosomes from cow milk for oral delivery. J Pharm Sci. (2019) 108:1496–505. 10.1016/j.xphs.2018.11.022 30468828 PMC6788294

[30] Zeng B Chen T Xie MY Luo JY He JJ Xi QY. Exploration of long noncoding RNA in bovine milk exosomes and their stability during digestion in vitro. J Dairy Sci. (2019) 102:6726–37. 10.3168/jds.2019-16257 31155266

[31] Li B Hock A Wu RY Minich A Botts SR Lee C. Bovine milk-derived exosomes enhance goblet cell activity and prevent the development of experimental necrotizing enterocolitis. PLoS ONE. (2019) 14:e0211431. 10.1371/journal.pone.0211431 30699187 PMC6353182

[32] Gao HN Guo HY Zhang H Xie XL Wen PC Ren FZ. Yak-milk-derived exosomes promote proliferation of intestinal epithelial cells in an hypoxic environment. J Dairy Sci. (2019) 102:985–96. 10.3168/jds.2018-14946 30580945

[33] Martin C Patel M Williams S Arora H Brawner K Sims B. Human breast milk-derived exosomes attenuate cell death in intestinal epithelial cells. Innate Immun. (2018) 24:278–84. 10.1177/1753425918785715 29991305 PMC6830917

[34] Manca S Upadhyaya B Mutai E Desaulniers AT Cederberg RA White BR. Milk exosomes are bioavailable and distinct microRNA cargos have unique tissue distribution patterns. Sci Rep. (2018) 8:11321. 10.1038/s41598-018-29780-1 30054561 PMC6063888

[35] Agrawal AK Aqil F Jeyabalan J Spencer WA Beck J Gachuki BW. Milk-derived exosomes for oral delivery of paclitaxel. Nanomedicine. (2017) 13:1627–36. 10.1016/j.nano.2017.03.001 28300659

[36] Samuel M Chisanga D Liem M Keerthikumar S Anand S Ang CS. Bovine milk-derived exosomes from colostrum are enriched with proteins implicated in immune response and growth. Sci Rep. (2017) 7:5933. 10.1038/s41598-017-06288-8 28725021 PMC5517456

[37] Vashisht M Rani P Onteru SK Singh D. Curcumin encapsulated in milk exosomes resists human digestion and possesses enhanced intestinal permeability *in vitro*. Appl Biochem Biotechnol. (2017) 183:993–1007. 10.1007/s12010-017-2478-4 28466459

[38] Hese IV Goossens K Vandaele L Opsomer G. Invited review: MicroRNAs in bovine colostrum-focus on their origin and potential health benefits for the calf. J Dairy Sci. (2020) 103:1–15. 10.3168/jds.2019-16959 31677833

[39] Izumi H Kosaka N Shimizu T Sekine K Ochiya T Takase M. Bovine milk contains microRNA and messenger RNA that are stable under degradative conditions. J Dairy Sci. (2012) 95:4831–41. 10.3168/jds.2012-5489 22916887

[40] Yamada T Inoshima Y Matsuda T Ishiguro N. Comparison of methods for isolating exosomes from bovine milk. J Vet Med Sci. (2012) 74:1523–5. 10.1292/jvms.12-0032 22785357

[41] Cheruvanky A Zhou H Pisitkun T Kopp JB Knepper MA Yuen PST. Rapid isolation of urinary exosomal biomarkers using a nanomembrane ultrafiltration concentrator. Am J Physiol Renal Physiol. (2007) 292:1657–61. 10.1152/ajprenal.00434.2006 17229675 PMC2271070

[42] Lai RC Arslan F Lee MM Sze NS Choo A Chen TS. Exosome secreted by MSC reduces myocardial ischemia/reperfusion injury. Stem Cell Res. (2010) 4:214–22. 10.1016/j.scr.2009.12.003 20138817

[43] Doyle LM Wang MZ. Overview of extracellular vesicles, their origin, composition, purpose, and methods for exosome isolation and analysis. Cells. (2019) 8:727. 10.3390/cells8070727 31311206 PMC6678302

[44] Conde-Vancells J Rodriguez-Suarez E Embade N Gil D Matthiesen R Valle M. Characterization and comprehensive proteome profiling of exosomes secreted by hepatocytes. J Proteome Res. (2008) 7:5157–66. 10.1021/pr8004887 19367702 PMC2696236

[45] Munagala R Aqil F Jeyabalan J Gupta RC. Bovine milk-derived exosomes for drug delivery. Cancer Lett. (2016) 371:48–61. 10.1016/j.canlet.2015.10.020 26604130 PMC4706492

[46] Benmoussa A Ly S Shan ST Laugier J Boilard E Gilbert C. A subset of extracellular vesicles carries the bulk of microRNAs in commercial dairy cow's milk. J Extracell Vesicles. (2017) 6:1401897. 10.1080/20013078.2017.1401897 29904572 PMC5994974

[47] Lee Y El Andaloussi S Wood MJ. Exosomes and microvesicles: extracellular vesicles for genetic information transfer and gene therapy. Hum Mol Genet. (2012) 21:R125–34. 10.1093/hmg/dds317 22872698

[48] Baietti MF Zhang Z Mortier E Melchior A Degeest G Geeraerts A. Syndecan-syntenin-ALIX regulates the biogenesis of exosomes. Nat Cell Biol. (2012) 14:677–85. 10.1038/ncb2502 22660413

[49] Mathivanan S Ji H Simpson RJ. Exosomes: extracellular organelles important in intercellular communication. J Proteomics. (2010) 73:1907–20. 10.1016/j.jprot.2010.06.006 20601276

[50] Tili E Michaille JJ Calin GA. Expression and function of micro-RNAs in immune cells during normal or disease state. Int J Med Sci. (2008) 5:73–9. 10.7150/ijms.5.73 18392144 PMC2288788

[51] Chen X Gao C Li H Huang L Sun Q Dong Y. Identification and characterization of microRNAs in raw milk during different periods of lactation, commercial fluid, and powdered milk products. Cell Res. (2010) 20:1128–37. 10.1038/cr.2010.80 20548333

[52] Admyre C Johansson SM Qazi KR Filen JJ Lahesmaa R Norman M. Exosomes with immune modulatory features are present in human breast milk. J Immunol. (2007) 179:1969–78. 10.4049/jimmunol.179.3.1969 17641064

[53] Lin D Chen T Xie M Li M Zeng B Sun R. Oral administration of bovine and porcine milk exosome alter miRNAs profiles in piglet serum. Sci Rep. (2020) 10:6983. 10.1038/s41598-020-63485-8 32332796 PMC7181743

[54] Gu Y Li M Wang T Liang Y Zhong Z Wang X. Lactation-related microRNA expression profiles of porcine breast milk exosomes. PLoS ONE. (2012) 7:e43691. 10.1371/journal.pone.0043691 22937080 PMC3427246

[55] van Herwijnen MJC Driedonks TAP Snoek BL Kroon AMT Kleinjan M Jorritsma R. Abundantly present miRNAs in milk-derived extracellular vesicles are conserved between mammals. Front Nutr. (2018) 5:81. 10.3389/fnut.2018.00081 30280098 PMC6153340

[56] Sun J Aswath K Schroeder SG Lippolis JD Reinhardt TA Sonstegard TS. MicroRNA expression profiles of bovine milk exosomes in response to Staphylococcus aureus infection. BMC Genomics. (2015) 16:806. 10.1186/s12864-015-2044-9 26475455 PMC4609085

[57] Wang M Zhao X Huang F Wang L Huang J Gong Z. Exosomal proteins: Key players mediating premetastatic niche formation and clinical implications (Review). Int J Oncol. (2021) 58:4. 10.3892/ijo.2021.5184 33649844 PMC7895540

[58] Sterzenbach U Putz U Low LH Silke J Tan SS Howitt J. Engineered exosomes as vehicles for biologically active proteins. Mol Ther. (2017) 25:1269–78. 10.1016/j.ymthe.2017.03.030 28412169 PMC5474961

[59] Vella LJ Hill AF Cheng L. Focus on extracellular vesicles: exosomes and their role in protein trafficking and biomarker potential in Alzheimer's and Parkinson's Disease. Int J Mol Sci. (2016) 17:173. 10.3390/ijms17020173 26861304 PMC4783907

[60] Rahman MM Takashima S Kamatari YO Badr Y Kitamura Y Shimizu K. Proteomic profiling of milk small extracellular vesicles from bovine leukemia virus-infected cattle. Sci Rep. (2021) 11:2951. 10.1038/s41598-021-82598-2 33536533 PMC7858626

[61] Izumi H Tsuda M Sato Y Kosaka N Ochiya T Iwamoto H. Bovine milk exosomes contain microRNA and mRNA and are taken up by human macrophages. J Dairy Sci. (2015) 98:2920–33. 10.3168/jds.2014-9076 25726110

[62] Kosaka N Izumi H Sekine K Ochiya T. microRNA as a new immune-regulatory agent in breast milk. Silence. (2010) 1:7. 10.1186/1758-907X-1-7 20226005 PMC2847997

[63] Pieters BC Arntz OJ Bennink MB Broeren MG van Caam AP Koenders MI. Commercial cow milk contains physically stable extracellular vesicles expressing immunoregulatory TGF-beta. PLoS ONE. (2015) 10:e0121123. 10.1371/journal.pone.0121123 25822997 PMC4379073

[64] Benmoussa A Lee CH Laffont B Savard P Laugier J Boilard E. Commercial dairy cow milk microRNAs resist digestion under simulated gastrointestinal tract conditions. J Nutr. (2016) 146:2206–15. 10.3945/jn.116.237651 27708120

[65] Leiferman A Shu J Upadhyaya B Cui J Zempleni J. Storage of extracellular vesicles in human milk, and microRNA profiles in human milk exosomes and infant formulas. J Pediatr Gastroenterol Nutr. (2019) 69:235–8. 10.1097/MPG.0000000000002363 31169664 PMC6658346

[66] Howard KM Jati Kusuma R Baier SR Friemel T Markham L Vanamala J. Loss of miRNAs during processing and storage of cow's (Bos taurus) milk. J Agric Food Chem. (2015) 63:588–92. 10.1021/jf505526w 25565082 PMC4387787

[67] Kirchner B Pfaffl MW Dumpler J von Mutius E Ege MJ. microRNA in native and processed cow's milk and its implication for the farm milk effect on asthma. J Allergy Clin Immunol. (2016) 137:1893–1895.e13. 10.1016/j.jaci.2015.10.028 26707195

[68] Baier SR Nguyen C Xie F Wood JR Zempleni J. MicroRNAs are absorbed in biologically meaningful amounts from nutritionally relevant doses of cow milk and affect gene expression in peripheral blood mononuclear cells, HEK-293 kidney cell cultures, mouse livers. J Nutr. (2014) 144:1495–500. 10.3945/jn.114.196436 25122645 PMC4162473

[69] Yu S Zhao Z Sun L Li P. Fermentation results in quantitative changes in milk-derived exosomes and different effects on cell growth and survival. J Agric Food Chem. (2017) 65:1220–8. 10.1021/acs.jafc.6b05002 28085261

[70] Ortega-Anaya J Jimenez-Flores R. Symposium review: the relevance of bovine milk phospholipids in human nutrition-Evidence of the effect on infant gut and brain development. J Dairy Sci. (2019) 102:2738–48. 10.3168/jds.2018-15342 30415849

[71] Chen T Xie MY Sun JJ Ye RS Cheng X Sun RP. Porcine milk-derived exosomes promote proliferation of intestinal epithelial cells. Sci Rep. (2016) 6:33862. 10.1038/srep33862 27646050 PMC5028765

[72] Ng PC Chan KY Leung KT Tam YH Ma TP Lam HS. Comparative MiRNA expressional profiles and molecular networks in human small bowel tissues of necrotizing enterocolitis and spontaneous intestinal perforation. PLoS ONE. (2015) 10:e0135737. 10.1371/journal.pone.0135737 26274503 PMC4537110

[73] Melnik BC Schmitz G. Exosomes of pasteurized milk: potential pathogens of Western diseases. J Transl Med. (2019) 17:3. 10.1186/s12967-018-1760-8 30602375 PMC6317263

[74] Chen Y Xiao Y Ge W Zhou K Wen J Yan W. miR-200b inhibits TGF-beta1-induced epithelial-mesenchymal transition and promotes growth of intestinal epithelial cells. Cell Death Dis. (2013) 4:e541. 10.1038/cddis.2013.22 23492772 PMC3613822

[75] Nata T Fujiya M Ueno N Moriichi K Konishi H Tanabe H. MicroRNA-146b improves intestinal injury in mouse colitis by activating nuclear factor-kappaB and improving epithelial barrier function. J Gene Med. (2013) 15:249–60. 10.1002/jgm.2717 23813877

[76] Good M Sodhi CP Egan CE. Breast milk protects against the development of necrotizing enterocolitis through inhibition of toll-like receptor 4 in the intestinal epithelium via activation of the epidermal growth factor receptor. Mucosal Immunol. (2015) 8:1166–79. 10.1038/mi.2015.30 25899687 PMC4540669

[77] Wu D Kittana H Shu J Kachman SD Cui J Ramer-Tait AE. Dietary Depletion of Milk Exosomes and Their MicroRNA Cargos Elicits a Depletion of miR-200a-3p and Elevated Intestinal Inflammation and Chemokine (C-X-C Motif) Ligand 9 Expression in Mdr1a^−/−^ Mice. Curr Dev Nutr. (2019) 3:nzz122. 10.1093/cdn/nzz122 32154493 PMC7053579

[78] Bartel DP. MicroRNAs: target recognition and regulatory functions. Cell. (2009) 136:215–33. 10.1016/j.cell.2009.01.002 19167326 PMC3794896

[79] Camussi G Deregibus MC Bruno S Cantaluppi V Biancone L. Exosomes/microvesicles as a mechanism of cell-to-cell communication. Kidney Int. (2010) 78:838–48. 10.1038/ki.2010.278 20703216

[80] Benmoussa A Gotti C Bourassa S Gilbert C Provost P. Identification of protein markers for extracellular vesicle (EV) subsets in cow's milk. J Proteomics. (2019) 192:78–88. 10.1016/j.jprot.2018.08.010 30153512

[81] Melnik BC Schmitz G. MicroRNAs: Milk's epigenetic regulators. Best Pract Res. Clin Endocrinol Metab. (2017) 31:427–42. 10.1016/j.beem.2017.10.003 29221571

[82] Naqvi AR Fordham JB Nares S. miR-24, miR-30b, and miR-142-3p regulate phagocytosis in myeloid inflammatory cells. J Immunol. (2015) 194:1916–27. 10.4049/jimmunol.1401893 25601927 PMC4323870

[83] Keller MD Ching KL Liang FX Dhabaria A Tam K Ueberheide BM. Decoy exosomes provide protection against bacterial toxins. Nature. (2020) 579:260–4. 10.1038/s41586-020-2066-6 32132711 PMC7519780

[84] Arntz OJ Pieters BC Oliveira MC Broeren MG Bennink MB de Vries M. Oral administration of bovine milk derived extracellular vesicles attenuates arthritis in two mouse models. Mol Nutr Food Res. (2015) 59:1701–12. 10.1002/mnfr.201500222 26047123

[85] Melnik BC John SM Schmitz G. Milk is not just food but most likely a genetic transfection system activating mTORC1 signaling for postnatal growth. Nutr J. (2013) 12:103. 10.1186/1475-2891-12-103 23883112 PMC3725179

[86] Lu Y Zheng Z Yuan Y Pathak JL Yang X Wang L. The emerging role of exosomes in oral squamous cell carcinoma. Front Cell Dev Biol. (2021) 9:628103. 10.3389/fcell.2021.628103 33718365 PMC7951141

[87] Duster R Prickaerts J Blokland A. Purinergic signaling and hippocampal long-term potentiation. Curr Neuropharmacol. (2014) 12:37–43. 10.2174/1570159X113119990045 24533014 PMC3915348

[88] Oliveira MC Arntz OJ Blaney Davidson EN van Lent PL Koenders MI van der Kraan PM. Milk extracellular vesicles accelerate osteoblastogenesis but impair bone matrix formation. J Nutr Biochem. (2016) 30:74–84. 10.1016/j.jnutbio.2015.11.017 27012623

[89] Lai RC Yeo RW Tan KH Lim SK. Exosomes for drug delivery - a novel application for the mesenchymal stem cell. Biotechnol Adv. (2013) 31:543–51. 10.1016/j.biotechadv.2012.08.008 22959595

[90] Sun D Zhuang X Xiang X Liu Y Zhang S Liu C. A novel nanoparticle drug delivery system: the anti-inflammatory activity of curcumin is enhanced when encapsulated in exosomes. Mol Ther. (2010) 18:1606–14. 10.1038/mt.2010.105 20571541 PMC2956928

[91] Somiya M Yoshioka Y Ochiya T. Biocompatibility of highly purified bovine milk-derived extracellular vesicles. J Extracell Vesicles. (2018) 7:1440132. 10.1080/20013078.2018.1440132 29511463 PMC5827637

[92] Vlassov AV Magdaleno S Setterquist R Conrad R. Exosomes: current knowledge of their composition, biological functions, and diagnostic and therapeutic potentials. Biochim Biophys Acta. (2012) 1820:940–8. 10.1016/j.bbagen.2012.03.017 22503788

[93] Zadoks RN Middleton JR McDougall S Katholm J Schukken YH. Molecular epidemiology of mastitis pathogens of dairy cattle and comparative relevance to humans. J Mammary Gland Biol Neoplasia. (2011) 16:357–72. 10.1007/s10911-011-9236-y 21968538 PMC3208832

[94] Cai M He H Jia X Chen S Wang J Shi Y. Genome-wide microRNA profiling of bovine milk-derived exosomes infected with Staphylococcus aureus. Cell Stress Chaperones. (2018) 23:663–72. 10.1007/s12192-018-0876-3 29383581 PMC6045547

[95] Kelleher SL Gagnon A Rivera OC Hicks SD Carney MC Alam S. Milk-derived miRNA profiles elucidate molecular pathways that underlie breast dysfunction in women with common genetic variants in SLC30A2. Sci Rep. (2019) 9:12686. 10.1038/s41598-019-48987-4 31481661 PMC6722070

[96] Feng YH Tsao CJ. Emerging role of microRNA-21 in cancer. Biomed Rep. (2016) 5:395–402. 10.3892/br.2016.747 27699004 PMC5038362

[97] Jiang J Yang P Guo Z Yang R Yang H Yang F. Overexpression of microRNA-21 strengthens stem cell-like characteristics in a hepatocellular carcinoma cell line. World J Surg Oncol. (2016) 14:278. 10.1186/s12957-016-1028-9 27793160 PMC5086074

[98] Teng Y Ren Y Sayed M Hu X Lei C Kumar A. Plant-derived exosomal microRNAs shape the gut microbiota. Cell Host Microbe. (2018) 24:637–52.e638. 10.1016/j.chom.2018.10.001 30449315 PMC6746408

[99] Zhou F Paz HA Sadri M Cui J Kachman SD Fernando SC. Dietary bovine milk exosomes elicit changes in bacterial communities in C57BL/6 mice. Am J Physiol Gastrointest Liver Physiol. (2019) 317:G618–24. 10.1152/ajpgi.00160.2019 31509432 PMC6879888

